# Prognostic immune markers for recurrence and survival in locally advanced esophageal adenocarcinoma

**DOI:** 10.18632/oncotarget.27052

**Published:** 2019-07-16

**Authors:** Laila Babar, Juliann E. Kosovec, Vida Jahangiri, Nobel Chowdhury, Ping Zheng, Ashten N. Omstead, Madison S. Salvitti, Matthew A. Smith, Ajay Goel, Ronan J. Kelly, Blair A. Jobe, Ali H. Zaidi

**Affiliations:** ^1^Esophageal and Lung Institute, Allegheny Health Network, Pittsburgh, PA, USA; ^2^Beckman Research Institute, City of Hope Comprehensive Cancer Center, Monrovia, CA, USA; ^3^Department of Hematology and Oncology, Charles A. Sammons Cancer Center, Baylor University Medical Center, Dallas, TX, USA

**Keywords:** esophageal adenocarcinoma, LAG3, IDO1, CXCL9, TIM3

## Abstract

Treatment options and risk stratification for esophageal adenocarcinomas (EAC) currently rely on pathological criteria such as tumor staging. However, with advancement in immune modulated treatments, there is a need for accurate predictive biomarkers that will help identify high-risk patients and provide novel therapeutic targets. Hence, we analyzed as prognostic classifiers a host of histopathological parameters in conjunction with novel immune biomarkers. Specifically, gene expression levels for CXCL9, IDO1, LAG3, and TIM3 were established in treatment naïve samples. Additionally, PD-L1 and CD8 positivity was determined by immunohistochemical staining. Based on our finding, a Cox model consisting of pathological complete response (CR), LAG3, and CXCL9 provided improved predictability for disease-free survival (DFS) compared to CR alone, and it demonstrated statistical significance for predictability of recurrence (p=0.0001). Likewise, for overall survival (OS), a Cox model constituted of TIM3, CR, and IDO1 performed better than CR alone, and it demonstrated statistical significance for predictability of survival (p = 0.0004). TIM3 was identified as the best predictor for OS (HR=4.43, p=0.0023). In conclusion, given the paucity of treatment options for EAC, evaluation of these biomarkers early in the disease course will lead to better risk stratification of patients and much needed alternatives for improved therapy.

## INTRODUCTION

Esophageal cancer is the 8th most common cancer worldwide, with esophageal adenocarcinoma (EAC) being the dominant subtype in the western hemisphere.[1] The prognosis of esophageal cancer is dismal, with an overall five-year survival rate of less than 20%.[2, 3] This rate falls even more dramatically for those patients presenting with metastatic disease, with a five-year survival less than 5%.[4] Currently in the United States, the preferred treatment for patients presenting with locally advanced EAC is neoadjuvant chemoradiation therapy (CRT) followed by surgical resection. Typically, 25-30% of these patients demonstrate a pathological complete response (CR) to neoadjuvant therapy, which is a proxy for favorable outcome, as this subset of responders has a five-year survival rate of approximately 60%.[5-7] On the contrary, the non-responders on this multimodality approach are subjected to unnecessary toxicity and delayed surgery with no apparent clinical benefit. Therefore, development of additional prognostic classifiers prior to initiation of neoadjuvant therapy is required to individualize treatment by helping to stratify treatment sensitive vs resistant patients. The utilization of CR on its own as a prognostic tool is mired in controversy, as there are not any universally agreed upon standards to capture survival benefit afforded to patients with a major but not complete pathological response.[8, 9]

Based on our current understanding of cancer biology, solid tumors are considered a heterogeneous collection of cancer cells with distinct differentiation, phenotypes, and functionality.[10] In addition to cancer cells, solid tumors are composed of multiple cellular components, including structural, endothelial, and immune cells.[11] It is well-established that cancer cells exert control on the non-malignant cell types in order to thrive and grow beyond the confines of the poorly vascularized microenvironment.[12] These cancer and stromal cells release immunosuppressive cytokines, such as IL-10 and TGF-β, to evade the immune system by establishing a pro-growth immune-resistant tumor microenvironment.[13] Additionally, as cancers cells grow and subclonal populations progress and differentiate, they accumulate non-silent point mutations through immune-editing that ultimately leads to loss of tumor immunogenic recognition.[14]

However, the immune response can still recognize and kill cancer cells through the release of chemokines and interferon signaling that leads to trafficking and infiltration of T-cells, primarily CD8+ cytotoxic cells, into the tumor compartments.[15] Unfortunately, tumors are able to develop tolerance to these secondary defenses through adaptive immune resistance, as they are able to evade antigen-specific T-cells by expression of ligands, such as PD-L1 and LAG3, which inactivate cytotoxic cells.[16, 17] This concept of adaptive resistance in T-cell inflamed phenotype cancers has been the foundation for successful development of efficacious immune checkpoint inhibitors for cancer therapy, where dormant intratumoral T-cells are activated by therapeutic blocking with antibodies, such as anti-PD-1, thereby reversing the immune escape mechanisms deployed by cancer cells. In a recent study, Kelly et al used immunohistochemistry (IHC) for CD8 and PD-L1 density scoring in primary gastroesophageal and gastric cancer patient specimens, where they demonstrated that higher CD8 densities were associated with higher PD-L1 expression, with the strongest staining reported in tumor-invasive fronts. Interestingly, patients with high CD8 and PD-L1 densities had poor progression-free survival (PFS) and overall survival (OS), suggesting a role of adaptive immune resistance mechanisms.[18]

In a recent publication, our group showed that in locally advanced EAC, the immune microenvironment is highly dynamic with a host of immune checkpoints being upregulated primarily post neoadjuvant CRT.[19] These immune escape mechanisms are not only therapeutically targetable for providing improved patient outcomes but can also be used to develop prognostic and predictive classifiers of response to specific therapies. There has been increasing evidence to suggest that activation of the anticancer immune responses are implicated in outcomes to even traditional therapies, such as cytotoxics and radiation.[20] Therefore, the purpose of this study is, 1) to determine degree of upregulation of immune biomarkers in treatment-naïve locally advanced EAC patients and 2) to develop robust models for prediction of disease recurrence and OS using combinations of independent histopathological parameters and individual immune biomarker classifiers.

## RESULTS

A total of 49 patients (48 male and 1 female) with locally advanced EAC who underwent chemotherapy +/- radiation therapy followed by esophagectomy were enrolled in the study. Mean age at diagnosis was 64.2 years (SD=9.5) with a median clinical follow up time of 21.2 months (IQR= 9.7, 30.3). As per the AJCC staging, 34 patients (69%) were staged as ≥T3, with the remaining staged as Table 1. Prior to surgery, 8 (16.3%) patients received chemotherapy only while 41 (83.7%) patients received chemotherapy + radiation therapy. Of these patients, 46 (93.9%) underwent minimally invasive esophagectomies while 3 (6.1%) had open esophagectomies. The resection margin was negative (R0) in 45 (92%), positive (R1) in 2 (4%) and unknown in 2 (4%) patients. Pathological response was observed in 39 (80%; CR 27%, partial response 53%), stable disease in 6 (12%) and progressive disease (distant metastasis) in 4 (8%) patients post-neoadjuvant therapy. Overall, 25 patients (51%) on this study developed a recurrence, and 29 patients (59%) are deceased - Table 1. Data for eight potential predictors (CR, clinical stage of tumor at the time of diagnosis, CD8 density, PD-L1 Status, CXCL9, IDO1, LAG3, and TIM3 is summarized with respect to recurrence and survival status, respectively - Table 2.

**Table 1:** Patient demographics

Total Number of Patients	49
**Gender**	
*Male*	98% (48)
*Female*	2% (1)
**Age (years)**	64.2 (9.5)
**Follow Up Time – (Months)**	21.2 (9.7, 30.3)
**Pathological Staging**	
* < T3*	31% (15)
*≥ T3*	69% (34)
**Pathological Response to Neoadjuvant Therapy**	
*Complete Response*	27% (13)
*Partial Response*	53% (26)
*Stable disease*	12% (6)
*Progression*	8% (4)
**Recurrence**	
*Yes*	51% (25)
*No*	49% (24)
**Survival Status**	
*Alive*	41% (20)
*Dead*	59% (29)
**Neoadjuvant Therapy**	
*Chemotheray Only*	16.3% (8)
*Chemotheray + Radiation Therapy*	83.7% (41)
**Type of Esophagectomy**	
*Minimally Invasive*	93.9% (46)
*Open*	6.1% (3)
**Resection Margin**	
*R0*	92% (45)
*R1*	4% (2)
*Unknown*	4% (2)
**Lymph Nodes Resected During Surgery**	
*1 - 5*	16.3% (8)
*6 – 10*	22.4% (11)
*11 – 15*	36.7% (18)
*16+*	20.4% (10)
*Unknown*	4.1% (2)

**Table 2:** Summary info for potential predictors for recurrence and survival

Potential Predictors	Recurrence		Survival Status	
	**No**	**Yes**	**Alive**	**Dead**
**Pathological Response**				
*No complete response*	15 (41.7%)	21 (58.3%)	13 (36.1%)	23 (63.9%)
*Complete response*	9 (69.2%)	4 (30.8%)	7 (53.9%)	6 (46.1%)
**Clinical Stage of Tumor**				
* < T3*	8 (53.3%)	7 (46.7%)	6 (40.0%)	9 (60.0%)
*≥ T3*	16 (47.1%)	18 (52.9%)	14 (41.2%)	20 (58.8%)
**PD-L1 Status**				
*Negative*	16 (51.6)	15 (48.4)	10 (32.3)	21 (67.7)
*Positive*	5 (45.4)	6 (54.6)	5 (45.5)	6 (54.5)
**CD8-Density**				
*Mean (SD)*	12.3 (12.5)	13.1 (14.2)	15.2 (14.4)	11.0 (12.4)
**CXCL9**				
*Mean (SD)*	2.1 (2.6)	1.4 (2.0)	1.7 (2.2)	1.8 (2.4)
**IDO1**				
*Mean (SD)*	2.1 (5.1)	3.1 (6.4)	1.1 (1.7)	3.6 (7.2)
**LAG3**				
*Mean (SD)*	1.9 (3.2)	2.8 (4.8)	1.2 (1.5)	3.1 (5.0)
**TIM3**				
*Mean (SD)*	4.4 (5.0)	5.1 (8.8)	2.8 (4.3)	6.1 (8.4)

Univariate analyses for each potential predictor with respect to DFS using Cox regression approach indicated a significant finding for CR (HR (95%CI) = 9.80 (2.20, 43.75), p = 0.0028), and borderline findings for CXCL9 at 0.2910 cut-off point (HR (95%CI) = 0.45 (0.19, 1.05), p = 0.0655) and LAG3 at 3.7521 cut-off point (HR (95%CI) = 2.29 (0.89, 5.89), p=0.0859). Univariate analyses for each potential predictor with respect to OS using Cox regression approach indicated significant findings for CR (HR (95%CI) = 3.92 (1.29, 11.93), p=0.0163), TIM3 at 3.9181 cut-off point (HR (95%CI) = 2.36 (1.11, 5.03), p=0.0264), and a borderline finding for LAG3 at 2.9759 cut-off point (HR (95%CI) = 2.09 (0.90, 4.82), p=0.0859)

Therefore, optimal RQ cut-off points were utilized to categorize the gene expression data with respect to time-to-event outcomes. To predict time-to-recurrence, the optimal RQ cut-off points for the individual genes were: CXCL9 (0.2910), IDO1 (2.0180), LAG3 (3.7521), TIM3 (3.0440). Likewise, to predict OS, the optimal RQ cut-off points were: CXCL9 (0.6506), IDO1 (0.2822), LAG3 (2.9759), TIM3 (3.9181). Next, utilizing these individual biomarker cut-off points, along with clinical classifiers, best fit multivariate Cox models for disease-free survival (DFS) and OS were generated.

For DFS, the optimal fitted model consisted of CR (HR=9.54, 95%CI=2.13-42.62, p=0.0032), LAG3 (HR=2.86, 95%=1.03-7.94, p=0.0441), and CXCL9 (HR=0.40, 95%CI=0.16-0.99, p=0.0494). Moreover, based on the –2 log likelihood statistic the classification performance of the CR/LAG3/CXCL9 Cox model (134.5) was superior to CR alone (140.7), as well as overall predictive accuracy in terms of Harrell's concordance statistics (0.7287 vs 0.6690), and the model demonstrated statistical significance for predictability of recurrence (p= 0.0001(LRT)). When adjusted for other covariates in the model, predicted times for months to recurrence were 45.6 months with CR, 27.8 months with LAG3 < 3.7521, and 28.2 months with CXCL9> 0.2910 – Table 3.

**Table 3:** Parameter estimates for each predictor adjusted for other covariates in the multivariate DFS Cox proportional hazard model

Predictor	Adjusted Predicted Time in Months to Recurrence	Estimate (SE)	Adjusted HR (95% CI)	P Value
**Pathological Response**				
*No complete response*	19.0	2.25 (0.76)	9.54 (2.13, 42.62)	0.0032
*Complete response (ref)*	45.6			
**LAG3**				
* < 3.7521 (ref)*	27.8	1.05 (0.52)	2.86 (1.03, 7.94)	0.0441
*≥ 3.7521*	17.4			
**CXCL9**				
* < 0.2910 (ref)*	19.0	-0.91 (0.46)	0.40 (0.16, 0.99)	0.0494
*≥ 0.2910*	28.2			

For OS, the optimal fitted model consisted of TIM3 (HR=4.43, 95%CI=1.70-11.53, p=0.0023) CR (HR=3.09, 95%CI=1.00-9.56, p=0.0505) and IDO1 (HR=0.31, 95%CI=0.11-0.82, p=0.0189). Likewise, the –2 log likelihood statistic demonstrated the classification performance of the TIM3/CR/IDO1 Cox model (164.7) was superior to CR alone (175.6), as well as overall predictive accuracy in terms of Harrell's concordance statistics (0.7106 vs 0.5945), and the model demonstrated statistical significance for predictability of OS (p=0.0004 (LRT)). When adjusted for other covariates in the model, predicted times for months to death were 50 months with CR, 44.8 months with TIM3 < 3.9181, and 43.4 months with IDO1 ≥ 0.2822 – Table 4.

**Table 4:** Parameter estimates for each predictor adjusted for other covariates in the multivariate OS Cox proportional hazard model

Predictor	Adjusted Predicted Time in Months to Death	Estimate (SE)	Adjusted HR (95% CI)	P Value
**Pathological Response**				
*No complete response*	26.7	1.13 (0.58)	3.09 (1.00, 9.56)	0.0505
*Complete response (ref)*	50.0			
**TIM3**				
* < 3.9181 (ref)*	44.8	1.49 (0.49)	4.43 (1.70, 11.53)	0.0023
*≥ 3.9181*	18.3			
**IDO1**				
* < 0.2822 (ref)*	21.8	-1.19 (0.51)	0.31 (0.11, 0.82)	0.0189
*≥ 0.2822*	43.4			

## DISCUSSION

The tumor immune microenvironment plays a dynamic role in the development, prognosis, and resistance of EAC to standard CRT therapeutics. Recent successes of anti-PD-1/PD-L1 immunotherapies across various malignancies have opened the door for further exploration into novel pathways that are similarly deregulated through cancer pathogenesis and contribute to tumor immune evasion.[21, 22] Specifically in the present study, we evaluated the prognostic significance of immune biomarkers such as CXCL9, IDO1, LAG3, and TIM3.

To date, CR post-neoadjuvant CRT has served as the best predictive clinical tool to determine PFS and OS benefit in local advanced EAC patients.[6, 7] The current National Comprehensive Cancer Network (NCCN) guidelines suggest that no evidence of disease in a post neoadjuvant esophagectomy specimen is the ideal marker to predict positive clinical outcomes.[26] Thus, CR was used as the control to evaluate the utility of our prediction models. Eight potential classifiers were studied including CR, clinical stage of tumor at the time of diagnosis, CD8 density, PDL-1 status, CXCL9, IDO1, LAG3, and TIM3. Univariate Cox regression analyses of these potential predictors showed statistical significance for CR and CR and Tim3 with DFS and OS, respectively and independently. Therefore, we determined optimal cut-off levels for each marker to more accurately determine causality and evaluate for collective prognostic significance.

The current study demonstrated that CR, LAG3, and CXCL9 were more predictive of DFS than CR alone and were significantly associated with reduced rate of recurrence. About 42% of esophageal cancer patients experience recurrence following complete surgical resection, despite pathologically-confirmed negative margins.[23] Additionally, the current standard of care approach for esophageal cancer, consisting of neoadjuvant CRT plus esophagectomy, is associated with considerable morbidity, so the reported panel may be beneficial in improving selection of patients that will likely benefit from such aggressive interventions. Additionally, LAG3 and CXCL9 pathways are under clinical investigation as novel targetable immune mechanisms; hence, patients with upregulation of these checkpoints may potentially benefit from tailored therapeutic modulation strategies. Specifically, LAG3 expression on CD8 T cells prevents autoimmunity; however, constitutive expression leads to immunoevasion through its negative regulatory role, in conjunction with PD-1.[24] In fact, there has been recent heightened interested in dual LAG3/PD-1 therapy due to this synergistic mechanism.[25] Of note, the efficacy of anti-PD1 in combination with anti-LAG is currently under clinical investigation in gastroesophageal cancer. Specifically, the phase Ib study, NCT03044613 is investigating neoadjuvant nivolumab +/- relatlimab in combination with CRT for locally advanced disease. Additionally, a phase Ib study, NCT03610711 has recently been launched to evaluate relatlimab in combination with nivolumab for the treatment of advanced gastroesophageal cancer. The other immune marker in this panel, CXCL9, is a chemokine that plays a complex role in tumor development and pathogenesis and not only recruits CD8 cytotoxic lymphocytes to inhibit tumor development, but also facilitates immune tolerance through recruitment of regulator T cells, tumor-associated macrophages, and myeloid derived suppressor cells.[26] CXCL9 has demonstrated both positive and negative prognostic values for various tumor types, including lung, breast, melanoma, gastric and renal cell carcinoma.[27] Additionally, CXCL9 is associated with regression of gastroesophageal cancer.[28] Most notably, its expression enhances the efficacy of various immunotherapies through regulation of targets, such as T cells, NK cells, APCs, and TILs.[27] As both LAG3 and CXCL9 pathways have a well demonstrated role in the tumor immune microenvironment, and the combination panel of CR, LAG3, and CXCL9 is significantly associated with prognosis, it may be of use to clinically explore their potential synergy with other immune checkpoints to possibly decrease the significant recurrence rates of EAC.

Likewise, our study demonstrated that CR, TIM3, and IDO1 were more predictive of OS than CR alone and significantly predicted survival. Moreover, TIM3 was the best individual predictor for OS (HR=4.43, 95% CI=1.70-11.53, p= 0.0023). As previously noted, only 25-30% of locally advanced EAC patients demonstrate a CR to neoadjuvant therapy, so this newly identified panel may better stratify patients to better inform therapeutic strategies most likely to lead to improved survival. Additionally, both TIM3 and IDO1 are under investigation as immune checkpoint inhibitors in solid tumors and may be worth exploring as alternative therapeutic strategies for locally advanced EAC, due to the significant dysregulation in association with prognosis. TIM3, like both PD1 and LAG3, is found on CD8 T cells, and increased expression has been implicated in immune evasion in various tumor types, including gastroesophageal cancer.[29-31] Currently, TIM3 inhibitors are being evaluated in early phase studies for various advanced solid tumors, which include esophageal cancer; however, there are no current studies specifically addressing esophageal or gastric malignancies, and preliminary results have not yet been reported.

The additional immunomarker in the presented panel, IDO1, is a tryptophan metabolizing catabolic enzyme that is activated in many malignancies and is usually associated with a poor prognostic outcome.[32] Mechanistically, IDO1 is immunosuppressive through inhibition of cytotoxic T cells and NK cells, while increasing the activity of regulatory T cells and myeloid-derived suppressor cells.[33] IDO1 also promotes angiogenesis.[34] Various agents targeting the IDO1 pathway are currently in clinical development, including indoximod (Phase 3), epacadostat (Phase 3), navozimod (Phase 1B), and BMS-986205 (Phase 2).[32] Indoximod demonstrated independent anti-tumor efficacy in various tumor types, and an early phase study has also revealed heightened efficacy when combined with anti-PD1 in melanoma patients, suggesting a potential role for combination therapy.[35] However, the recent phase 3 ECHO-301 trial evaluated epacadostat in combination with pembrolizumab in advanced melanoma showed no increased benefit. A retrospective evaluation suggests the trial design may not have provided sufficient drug exposure, and further studies exploring alternative agents are warranted.[36] Due to the very poor survival outcomes of EAC patients and synergistic immunomodulatory nature of both TIM3 and IDO1, it is possible that dual anti-TIM3/anti-IDO1 combination therapies may have an impact on survival for EAC patients; however, extensive clinical studies would be required to investigate further.

A notable limitation to the current study was a small sample size. However, CR independently demonstrated significant correlation with DFS and OS consistent with previously reported studies, and the specific models discussed in this manuscript demonstrated improved prognostic value when compared to CR alone. Additionally, routine sampling depth achieved through endoscopic biopsy may result in marginal access to the highly immunogenic tumor invasive front, so the reported levels of the sampled tissue may not fully characterize the entire mass. Therefore, it may be of interest to explore emerging non-invasive liquid biopsy approaches to fully characterize the immune profile of the tumor, more selectively personalize therapeutics, and evaluate response and molecular marker levels throughout treatment.

In conclusion, EAC is a deadly disease with poor progression-free survival rates, and current standard of care approaches are associated with significant morbidity. Although CR has historically been the best prognostic indicator, about 40% of patients still experience recurrence, and the five-year survival rate is only 60%. The present study evaluated the prognostic value of select immune checkpoints and identified novel predictive panels that better predict recurrence and survival than CR alone. Additionally, all of the immunomarkers identified in this study are currently under development as novel immunotherapeutic strategies, and further studies may be warranted to determine if the associated pathways may be modulated to improve clinical outcomes in selected patients.

## MATERIALS AND METHODS

### Clinical ethics statement

The retrospective study and a waiver of informed consent were approved by the Institutional Review Board at Allegheny Health Network in Pittsburgh, PA, under Protocol #16–002: Analysis of immune biomarkers for the treatment of locally advanced esophageal cancer. All patient samples were collected from tissues that remained post diagnostic pathology. The sample set utilized included formalin-fixed paraffin-embedded (FFPE) cases of normal esophageal epithelium (n = 15) and treatment naive endoscopic biopsies (prior to neoadjuvant CRT) from locally advanced EAC cases (n = 49). Additionally, deidentified demographic and clinical outcome data were collected on all patients.

### Laser capture microdissection and gene expression

FFPE samples were reviewed by a board-certified pathologist to identify areas of EAC. Briefly, laser capture microdissection (LCM) was performed on all samples to collect tumor epithelial cells, tumor infiltrating lymphocytes (CD8), and stromal myeloid cells, as reported previously.[37] Total RNA, containing miRNA, was isolated from post-LCM tissues. RNA was reverse transcribed, and preamplification was performed on cDNAs per manufacturer’s guidelines.[37] RT-PCR was performed according to the manufacturer’s guidelines using the following RT^2^ Primer Assays: TIM3 (Qiagen, Valencia, CA #PPH00583A), IDO1 (Qiagen, Valencia, CA #PPH01328B), LAG3 (Qiagen, Valencia, CA #PPH01035A) CXCL9 (Qiagen, Valencia, CA # PPH00700B). Relative gene expression was calculated using the ΔΔ-Ct method, and presented as relative quantification (RQ). SNORD95 and miR-16 were selected as endogenous controls, and RTC was run as a quality control on each plate. Gene expression for tumor samples was performed and normalized against pathologically confirmed LCM normal esophagus samples. All experiments were performed in technical duplicates.

### Immunohistochemistry

The FFPE EAC samples were stained for PD-L1 and CD8 using 22C3 immunohistochemistry (IHC) pharmDx (Dako, Carpinteria, CA; #SK00621) and CD8 antibody (Thermo Fisher Scientific, Waltham, MA; #RB-9009-P0), respectively. Briefly, 4 to 5.6mm tissue sections were placed onto positively charged slides using a microtome. IHC was performed using a Ventana BenchMark ULTRA automated stainer (Ventana, Tucson, AZ; #N750-BMKU-FS). PD-L1 was supplied prediluted and applied. The CD8 antibody was applied at a dilution of 1:50. The slides were stained using standard clinically established automated protocols. PD-L1 stains were deemed positive based on >1% staining for tumor or lymphocytic cells. Additionally, intensity was gauged based on the magnification required to visualize positive cells, defined as weak (20x), moderate (10x), and strong (4x).For CD8 positivity, a quantitative density analysis was performed for each sample using 3 selected microscopic fields and counting the number of stained CD8 cells per 100 tumor cells.[38] Tonsil tissue and provided tumor control tissue served as positive controls for CD8 and PD-L1, respectively. Scoring was performed by a blinded board certified pathologist.

### Statistical analysis

Eight variables (CR status post neoadjuvant CRT, clinical stage of tumor at the time of diagnosis, PD-L1 positivity, CD8 positivity, and gene expression levels for CXCL9, IDO1, LAG3, and TIM3) were selected to evaluate their prognostic significance in predicting recurrence and survival status.

Univariate Cox regression analyses were performed for each of 8 potential predictors, utilizing continuous and categorical cut-off points, with respect to each of the time-to-event outcome. Next, we developed a multivariate Cox’s proportional hazards model for each time-to-event outcome, utilizing a stepwise selection that mandated a variable has to have a significance threshold of 0.25 and 0.10 to be opted and retained in the model, respectively. The final models selected demonstrated superior overall predictive accuracy and model fit statistics. In particular, an optimal fitted model will have the lowest value of –2 log likelihood statistic with Likelihood Ratio Test significant (p < 0.05) to indicate an alternative assumption that at least one explanatory effect in the model is not zero; moreover, overall predictive accuracy provided by Harrell's concordance statistic has largest value, which measures the agreement between observed and predicted outcomes. In our study, a statistically significant association between a predictor and the time-to-event outcome is only established if p-value < 0.05 for Type 3 Wald Test or the 95% hazard ratio confidence interval does not cross 1.0.

Specifically, for gene expression data we utilized both a continuous and a categorical optimal cut-off point input approach to develop candidate models for each of the time-to-event outcomes. In particular, to determine the optimal cut-off point for each gene, an outcome-oriented approach proposed by Contal and O’Quigley was utilized to categorize the gene expression levels that enhance the absolute value of log-rank test statistic, hence capturing the maximum difference for subjects in the two groups.[39]

All statistical analyses on the study were performed using SAS software (version 9.4; SAS Institute, Cary, NC).

## Abbreviations

EAC: Esophageal adenocarcinomas
CR: complete response
DFS: disease-free survival
CRT: chemoradiation therapy
OS: overall survival
PFS: progression-free survival
NCCN: National Comprehensive Cancer Network
FFPE: formalin-fixed paraffin-embedded
LCM: laser capture microdissection
RQ: relative quantification
IHC: immunohistochemistry
